# Serum markers of type III and IV procollagen processing predict recurrence of fibrosis in liver transplanted patients

**DOI:** 10.1038/s41598-019-51394-4

**Published:** 2019-10-16

**Authors:** Mette Juul Nielsen, Ida Falk Villesen, Natasja Stæhr Gudmann, Diana Julie Leeming, Aleksander Krag, Morten Asser Karsdal, Tim Zimmermann, Detlef Schuppan

**Affiliations:** 1 Nordic Bioscience, Fibrosis Biology and Biomarkers, Herlev, Denmark; 2 Department of Medicine I, Transplant Hepatology, University Medical Center, Mainz, Germany; 3 Department of Gastroenterology and Hepatology, Odense University Hospital, University of Southern Denmark, Odense, Denmark; 4 Institute of Translational Immunology, University Medical Center, Mainz, Germany; 5 Division of Gastroenterology, Beth Israel Deaconess Medical Center, Harvard Medical School, Boston, MA USA

**Keywords:** Diagnostic markers, Predictive markers, Prognostic markers

## Abstract

Following liver transplantation (LT), 10–30% of patients develop recurrent cirrhosis (RC). There is an urgent need for predictive non-invasive markers for improved monitoring of these patients. Here we studied extracellular matrix biomarkers as predictors of RC after LT. Forty-seven LT patients were divided into groups of fast, intermediate or non-progressors towards RC (<1 year, 3–5 years or no advanced fibrosis >5 years after LT), assessed by follow-up liver biopsies. Markers of interstitial matrix type III and V collagen formation (PRO-C3 and PRO-C5), basement membrane type IV collagen formation (PRO-C4) and degradation (C4M) were assessed in serum samples collected 3, 6 and 12 months post-LT using specific ELISAs. PRO-C3, PRO-C4, and C4M were elevated in fast progressors compared to non-progressors 3 months after LT. C4M and PRO-C4 additionally differentiated between intermediate and fast progressors at 3 months. PRO-C3 was best predictor of survival, with LT patients in the highest PRO-C3 tertile having significantly shorter survival time. This shows that interstitial matrix and basement membrane remodeling in RC may be distinguishable. Markers originating from different sites in the extracellular matrix could be valuable tools for a more dynamic monitoring of patients at risk of RC. However, this needs validation in larger cohorts.

## Introduction

Liver transplantation (LT) is the last therapeutic option for patients with end-stage liver disease. Until recently, before the advent of highly effective antiviral therapies, recurrent hepatitis C infection has been the most common cause of rapid post-LT fibrosis progression^1^, graft loss and mortality within 1 to 10 years after LT^2^. Other liver diseases also recur after transplantation with incidence rates ranging from 10% to 50% including alcoholic liver disease (ALD)^3^, primary sclerosing cholangitis (PSC)^4^, primary biliary cholangitis (PBC)^4^, autoimmune hepatitis (AIH)^4^ and non-alcoholic steatohepatitis (NASH)^5^. Fibrosis is the result of accelerated accumulation of extracellular matrix (ECM) proteins, in particular interstitial types I, III, and V collagens that increase up to 6-fold in advanced liver fibrosis^6^. The prominent basement membrane type IV collagen is also prone to substantial remodeling especially during early liver fibrosis and can be increased up to 10-fold^7^. It has been suggested that basement membrane remodeling, as seen in fibrosis, is largely driven by liver epithelial cells in an attempt to regenerate the ECM as an initial repair response^8,9^.

By assessing specific fragments of collagens generated by proteases, it should be possible to separate tissue formation vs degradation. Hence, we have developed a panel of serological biomarkers using the Protein Fingerprint Technology™, to quantify the tissue balance. Combining disease relevant proteases and up-regulated proteins of fibrogenesis, results in generation of a fingerprint specific for the affected tissue. The (pro-)collagen fragments are released from the tissue into the circulation, where they can be identified by neo-epitope specific ELISAs to permit evaluation of the ECM remodeling during liver fibrosis, and potentially serve as prognostic biomarkers for progression to cirrhosis^10^. Measurements of these neo-epitopes have previously proved to be more sensitive and accurate than routinely used diagnostic and prognostic tools^11–13^.

Type III and type V collagens are important components of the reticular fibers generated by (myo-)fibroblasts in the interstitial matrix^14^, which is the main local area affected by inflammation^15^. Maturation of type III and V collagen includes cleaving off the N- and C-terminal pro-peptide by specific proteases^14^. In fibrotic liver diseases, release of these pro-peptides, i.e. PRO-C3 and PRO-C5, can be highly increased^13,16,17^.

We have previously suggested that the marker of central tetrameric crosslinking domain of type IV collagen, PRO-C4, reflects enhanced basement membrane synthesis and turnover during accelerated ECM remodeling in liver fibrosis^8,18,19^. Furthermore, a marker of matrix metalloproteinase (MMP)-mediated type IV collagen degradation, C4M, reflects unfavorable basement membrane degradation^20^. Together these two markers may serve as a tool for monitoring unfavorable basement membrane turnover in liver fibrogenesis^19^.

Development and progression of recurrent liver fibrosis may follow different paths dependent on the underlying etiology. Even though the etiologies are different, the end-stage disease is comparable, i.e. advanced fibrotic and architectural remodeling in the transplanted liver, leading to graft loss and death. Early identification of patients at risk of rapid liver fibrosis progression could enable life-saving interventions with timely intervention, e.g., changes in immunosuppressive regimens or even antifibrotic agents that are in development^21,22^. Here we investigated the prognostic utility of four Protein Fingerprint serological biomarkers.

## Results

Demographics are presented in Table 1. Eleven patients developed cirrhosis within 1 year after LT and 19 within 3–5 years. Another 17 patients showed no or mild fibrosis over the first 5 years post-LT.

**Table 1:** Patient characteristics divided into progressor groups.

Post-LT cirrhosis progression rate	Fast	Intermediate	None	p-value
	(≤1 year)	(3–5 years)	(no cirrhosis)	
Gender, male/n (%)*	8/11 (73%)	12/19 (63%)	13/17 (76%)	0.669
Mean age, yr [95%CI]**	55.7 [52.2–59.1]	54.4 [50.1–58.8]	49.8 [43.7–56.0]	0.218
Mean graft survival time, yr [95% CI]**	5.2 [2.7–7.7]	8.4 [6.6–10.2]	12.9 [12.0–13.8]	<0.001
HCV positive*	4 (36%)	9 (47%)	8 (47%)	0.833
HCC*	3 (27%)	5 (26%)	5 (29%)	0.979
**Aetiology of CLD, n (%)** **:**				
Viral (HBV, HCV)	4 (36%)	11 (58%)	7 (41%)	
ALD	5 (46%)	3 (16%)	2 (12%)	
Autoimmune (AIH, PSC, PBC)	1 (9%)	4 (21%)	4 (24%)	
Other	1 (9%)	1 (5%)	4 (24%)	
**Cause of recurrent fibrosis (n)** **:**				
Viral (HCV/HBV)	4/0	11/1		
Biliary	3	3		
Immune mediated	4	3		
Alcohol	0	1		
Bilirubin (at baseline, mean [95% CI])**	2.3 [0.6–3.9]	2.2 [0.7–3.6]	0.9 [0.7–1.0]	0.003
Rejection episodes*	5 (46%)	6 (32%)	5 (29%)	0.659
Bile duct complications*	5 (46%)	12 (63%)	6 (35%)	0.247
Tac*	10 (91%)	15 (79%)	15 (88%)	0.615
**Outcome, n (%)** **:**				
Alive	2 (18%)	6 (32%)	15 (88%)	
Re-LT	1 (9%)	4 (21%)	0 (0%)	
Dead	8 (73%)	9 (47%)	2 (12%)	

LT: Liver transplantation; HCV: Hepatitis C infection; HCC: Hepatocellular carcinoma; CLD: Chronic liver disease; HBV: Hepatitis B infection; ALD: Alcoholic liver disease; AIH: Autoimmune hepatitis; PSC: Primary sclerosing cholangitis; PBC: Primary biliary cirrhosis; ALT: Alanine aminotransferase; Tac: Tacrolimus; CSA: Cyclosporine A. Data are shown as mean with 95% CI for continuous variables and as total number (%) for categorical variables. *P-values calculated by Χ^2^-test, **P-values calculated by One-way ANOVA.

Most patients were male with similar mean age (p = 0.218). Mean survival time after transplantation was 5 years in the fast progressor group, whereas patients with no or mild signs of fibrosis after 5 years had a mean survival time of 13 years (p < 0.001); data not shown. At the end of the study period, only 18% of patients in the fast progressor group were alive compared with 32% in the intermediate progressor group, and 88% in the non-progressor group.

**Figure 1:**
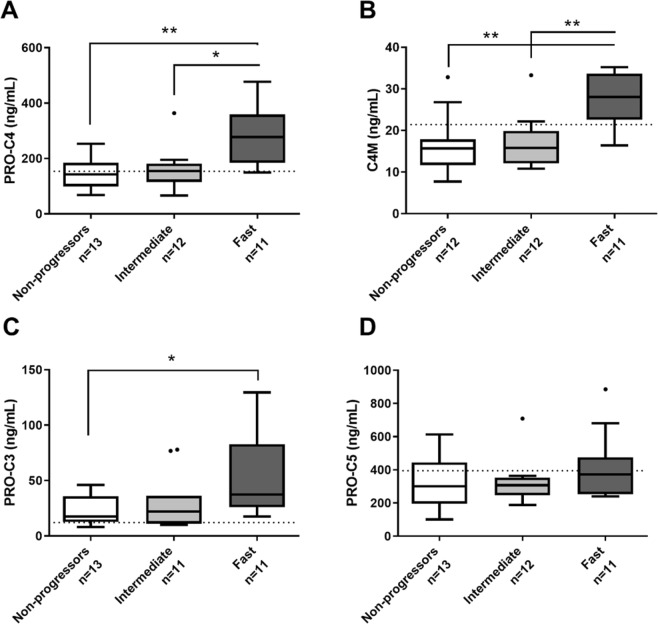
Tukey Boxplots of baseline biomarker concentrations for PRO-C4 (**A**), C4M (**B**), PRO-C3 (**C**), and PRO-C5 (**D**). Patients were stratified according to their progression rate towards cirrhosis after LT.

At baseline (3 months post-LT), serum levels of PRO-C3, PRO-C4 and C4M were significantly elevated in fast progressors compared to non-progressors (p < 0.05–p < 0.01) (Fig. 1A–C). No difference was observed for PRO-C5 (Fig. 1D). Interestingly, markers of type IV collagen formation and degradation (PRO-C4 and C4M), in contrast to markers of type III and V collagen formation (PRO-C3 and PRO-C5) could additionally distinguishing between the two progressor groups, p < 0.05 and p < 0.01, respectively (Fig. 1A,B).

**Figure 2:**
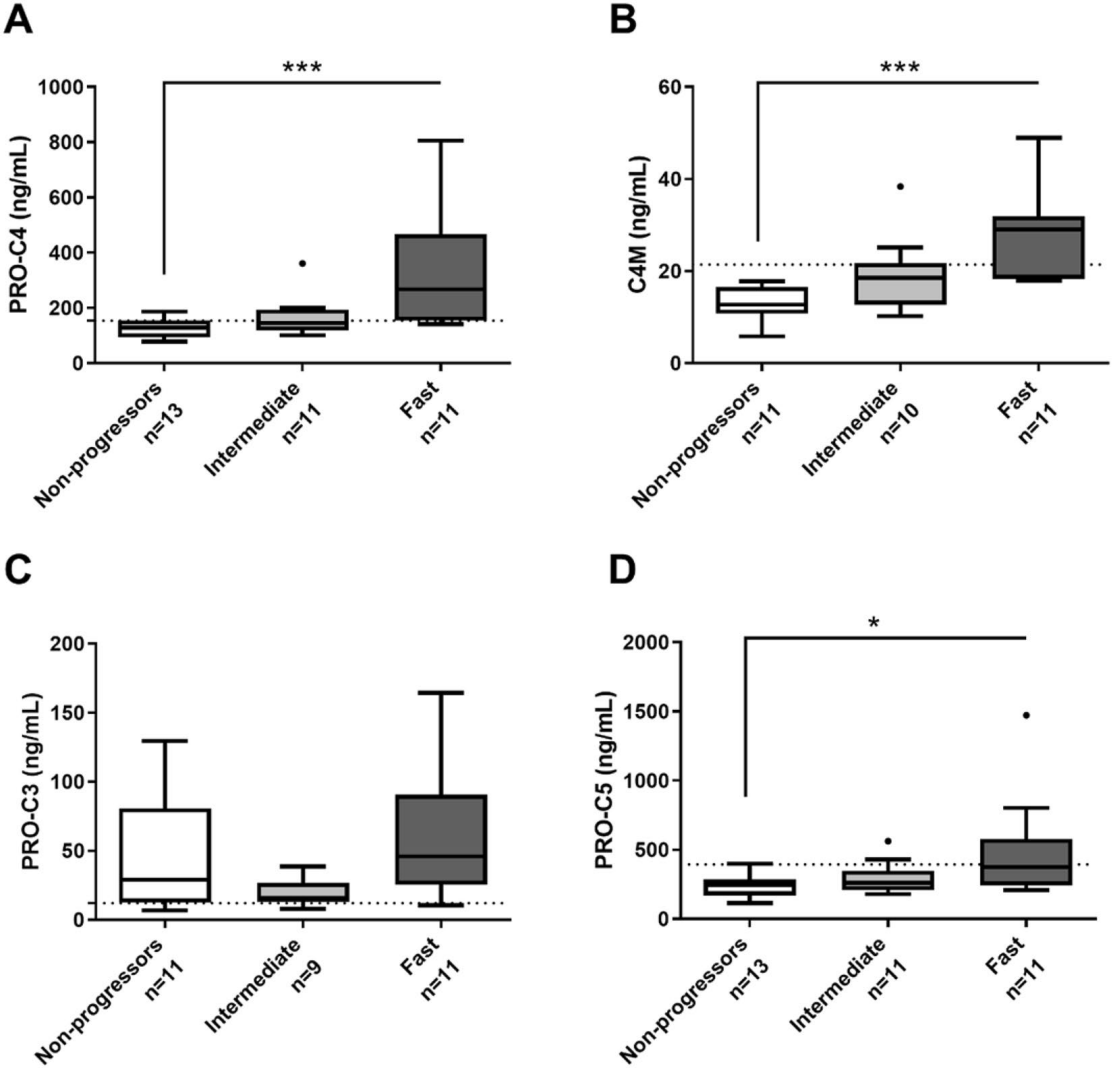
Tukey Boxplots of biomarker concentrations 6 months post-LT for PRO-C4 (**A**), C4M (**B**), PRO-C3 (**C**), and PRO-C5 (**D**). Patients were stratified according to their progression rate towards cirrhosis after LT.

In addition, serum levels of PRO-C4 and C4M remained elevated at 6 months post-LT, in fast progressors compared to non-progressors (p < 0.001) (Fig. 2A,B) thus continuing the trend of increased biomarker levels. PRO-C5 levels at 6 months post-LT were similarly found to be increased in fast- compared to non-progressors (p < 0.05), whereas no difference was found for PRO-C3 (Fig. 2C,D). When investigating the association between clinical parameters and biomarkers one year after transplantation, tissue inhibitors of metalloproteinase-1 (TIMP-1) was significantly correlated to PRO-C3, PRO-C4, PRO-C5 and C4M. In addition, bilirubin, and ELF, including all three components, were significantly correlated to PRO-C3 (Supplementary Table 2).

**Figure 3:**
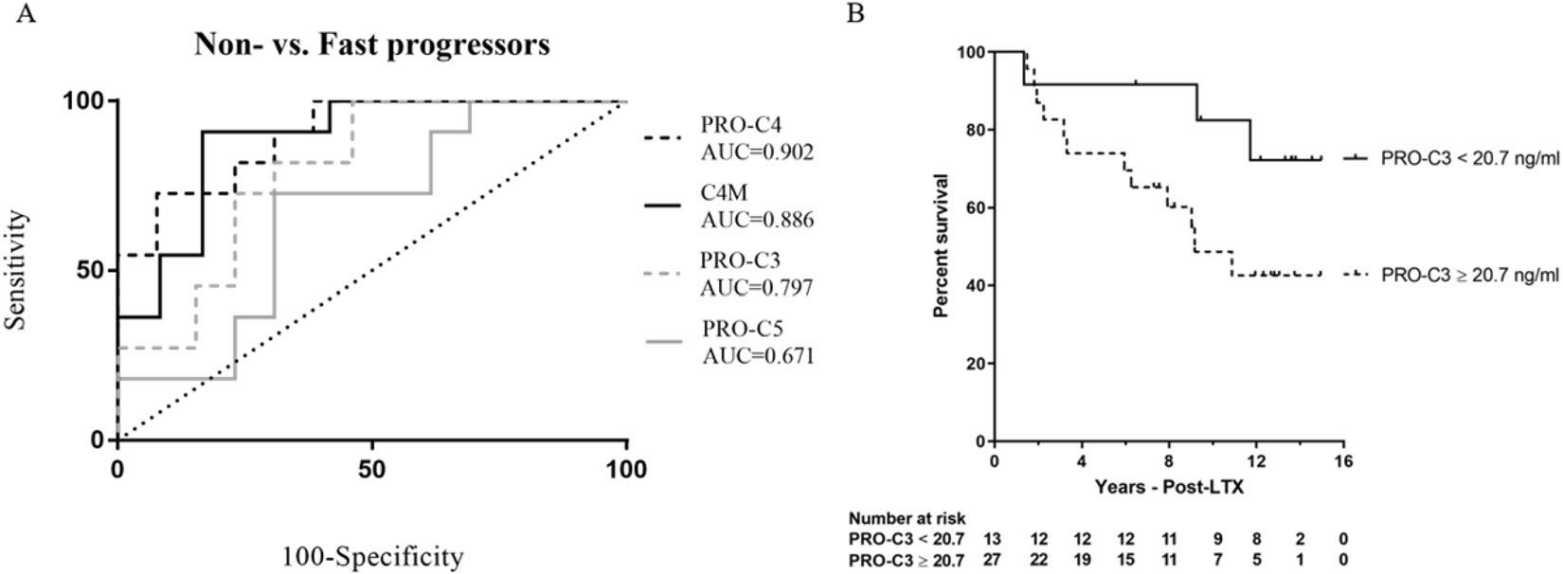
Predictive value of PRO-C4, C4M, PRO-C3 and PRO-C5. (**A**) ROC curve discriminating non- from fast-progressors towards cirrhosis. N = 11–13 per group. (**B**) Survival of patients with progression towards cirrhosis after transplantation. All patients were divided into two groups according to serum levels of PRO-C3. Baseline values of PRO-C3 < 20.7 (N = 13), and PRO-C3 ≥ 20.7 (N = 27) were compared as predictors of survival.

ROC curve for separating fast- from non-progressors further confirmed PRO-C4, C4M, and PRO-C3 as early predictors of fast progressors. No significant ROC curve was found for PRO-C5 (Fig. 3A).

Additionally, AUROC analyses found PRO-C4 and C4M to be the best markers for detecting fast progressors towards cirrhosis from non-progressors at baseline with AUCs of 0.90 [95% CI 0.7–0.9] and 0.88 [95% CI 0.6–0.9], and specificities >80% (Table 2). PRO-C3 predicted fast progressors with AUC = 0.79 [95% CI 0.5–0.9], sensitivity of 100%, and with specificity of 54%. PRO-C5 performed only moderate with AUC = 0.67 [95% CI 0.4–0.8], specificity of 69% and sensitivity of 72%. When separating intermediate and fast progressors, PRO-C4 and C4M were superior to PRO-C3 and PRO-C5 with AUCs > 0.85 [95% CI 0.6–0.9] and specificity of 75% and 83% respectively and sensitivity of 91% for both. However, none of the markers could distinguish between intermediate and non-progressors.

**Table 2:** Performance of serum markers 3 months post-LT to discriminate between intermediate and fast progressors, and the non-progressors towards cirrhosis.

	Marker	AUC [95% CI]	Cut-off	Sens	Spec	+LR	−LR	p-value
Fast vs. Non-progressors	PRO-C4	0.90[0.71–0.98]	>218	72	92	9.4	0.3	<***0.001***
	C4M	0.88[0.68–0.98]	>18	90	83	5.4	0.1	<***0.001***
	PRO-C3	0.79[0.58–0.93]	>17	100	54	2.1	0.0	***0.0014***
	PRO-C5	0.67[0.45–0.84]	>340	72	69	2.3	0.3	0.137
Intermediate vs. Fast-progressors	PRO-C4	0.86[0.65–0.97]	>175	91	75	3.6	0.1	<***0.001***
	C4M	0.90[0.71–0.98]	>20	91	83	5.4	0.1	<***0.001***
	PRO-C3	0.77[0.55–0.92]	>16	100	45	1.8	0.0	***0.007***
	PRO-C5	0.70[0.48–0.87]	>354	63	83	3.8	0.4	0.086
Intermediate vs. Non-progressors	PRO-C4	0.57[0.35–0.76]	>101	91	38	1.4	0.2	0.560
	C4M	0.54[0.33–0.75]	>18	41	83	2.5	0.7	0.696
	PRO-C3	0.53[0.32–0.74]	>34	27	76	1.1	0.9	0.758
	PRO-C5	0.53[0.33–0.73]	>340	41	69	1.3	0.8	0.753

AUC: Area under the ROC curve; Sens: Sensitivity; Spec: Specificity; ± LR: positive and negative likelihood ratios.

The cutoff (ng/ml) is determined from the ROC analyses giving the highest sensitivity and specificity.

To evaluate the prognostic utility of the markers, the patients were stratified into tertiles in accordance to the baseline level of each marker. Baseline levels of C4M and PRO-C4 were able to predict progression towards cirrhosis after transplantation. Patients in the highest C4M tertile (T3) had a 21-fold [95% CI 1.5–293.2] higher risk of recurrent cirrhosis compared with the patients in the lowest tertile (T1) (p < 0.05). Similarly, baseline PRO-C4 in patients of T3 predicted progression with an OR of 85 [95% CI 3–2417] compared with T1 (p < 0.01) (Table 3).

**Table 3:** Prognostic power of C4M, PRO-C4, PRO-C3 and PRO-C5 to predict progression towards cirrhosis after liver transplantation.

Non- vs. fast-progressors	Total no. of patients	No. of progressors	OR [95% CI]	P-value	OR [95% CI] for being progressors
C4M T1	7	1	1		
C4M T2	8	4	9.3 [0.7–122.5]	0.089	
C4M T3	7	6	21 [1.5–293.2]	**0.024**	
PRO-C4 T1	8	0	1		
PRO-C4 T2	7	4	21.9 [0.9–523]	0.057	
PRO-C4 T3	8	7	85 [3–2417]	**0.009**	
PRO-C3 T1	7	0	1		
PRO-C3 T2	8	5	23.6 [1–556.1]	0.050	
PRO-C3 T3	9	6	27.9 [1.2–646.1]	**0.038**	
PRO-C5 T1	8	3	1		
PRO-C5 T2	7	3	1.3 [0.1–9.9]	0.833	
PRO-C5 T3	8	5	2.7 [0.3–21]	0.323	

Patients were stratified according to tertiles of biomarker serum levels at 3 months. The lowest tertile (T1) was used as a reference when calculating odds ratio of being progressor for T2 and T3. The total number of patients as well as number of progressors in each tertile are shown.

In multivariate Cox regression analysis of baseline measurements, PRO-C3 was independently associated with survival. Kaplan-Meier analysis, using a cut-off value = 20.7 ng/mL defined from the Youden index that was derived from ROC curve analysis, was used to investigate the association of PRO-C3 and survival. Mean survival was significantly shorter in patients with high PRO-C3 levels compared to patients with low PRO-C3 levels (Mean age 9.3 vs 13.1 years, respectively, p < 0.05) (Fig. 3B).

## Discussion

Rapid recurrent cirrhosis after LT occurs in subjects with viral and non-viral etiologies of end-stage liver disease^1,3–5^. Despite advances in surgical techniques and periprocedural medical therapy, including effective therapies to eliminate HCV infection, there remains a high risk due to immune suppression and/or recurrent non-HCV liver disease^1,5^. Thus, the need to noninvasively monitor patients with risk of progression after LT is an urgent need as repeated biopsies is associated with patient discomfort and complications, and do not reflect the molecular dynamics of fibrosis progression^21–23^. Here we investigated the prognostic utility of four defined ECM remodeling markers as non-invasive tools to identify patients at risk of rapid liver fibrosis progression. Irrespective of progressor group, the cause of initial fibrosis and cirrhosis was either viral or non-viral, supporting that RC occurs in non-viral LT patients. The main findings of the study were: (1) Three months post-LT, PRO-C4, C4M and PRO-C3 differentiated between patients with fast progression towards RC vs non-progression; (2) PRO-C4 and C4M could discriminate intermediate progressors from fast progressors towards RC; and (3) PRO-C3 was independent predictor of post-LT survival.

Elevated serum levels three months post-LT in fast progressors indicate that ECM turnover can be detected earlier by the serological biomarkers before histological evaluation show advanced fibrosis or cirrhosis, thus enabling identification of a fast fibrosis progression endotype and early recognition of patients in most need of regular monitoring and possibly urgent treatment.

Some patients are more prone to fibrosis progression than others after LT, and apart from some early clinical indicators such as repetitive severe rejection episodes, predictive markers are lacking^24^. Interestingly, markers associated with formation and degradation of basement membrane collagen (PRO-C4 and C4M, respectively) and interstitial collagen formation (PRO-C3) were elevated already 3 months after transplantation, whereas a marker of type V collagen formation, PRO-C5, related to interstitial collagen fibril formation seemed to increase only with a delay in the fast progressors. This could indicate that unfavorable liver collagen turnover and unbalanced remodeling are operative immediately after transplantation due to factors related to the host, donor, or surgical procedure^25^, whereas other specialized ECM structures (e.g. type V procollagen) are established later, possible also due to co-morbidities associated with end-stage liver disease, regardless of primary cause of chronic liver disease.

PRO-C3 was not only independent predictor of RC, but patients with high baseline PRO-C3 levels had significantly shorter survival time after transplantation than patients with lower PRO-C3 levels, indicating that PRO-C3 reflects both higher risk of RC and survival. This has important clinical implications since it has potential to provide early diagnostic and prognostic information and thus help guide clinical decision-making and early institution of potential antifibrotic therapies^1,21,22^. Moreover, by using serum markers, the efficacy of antifibrotic intervention with possible positive effects in the immunosuppressive regimen, may be detectable after only a few days or weeks. However, these results need to be validated in larger intervention cohorts.

With this study, we showed that patients with high type IV collagen turnover after LT had a up to 85-fold increased risk of RC compared to patients with low levels. The type IV collagen formation marker PRO-C4 was best at separating non- from fast-progressors towards RC with an AUC = 0.90 with sensitivity and specificity of 72% and 92%, respectively. PRO-C4 has previously been shown to be associated with liver fibrosis^18^ but this is the first study showing its prognostic potential. The robustness of PRO-C4 and C4M as prognostic markers of clinical outcome needs to be investigated in larger cohorts.

PRO-C3 has previously been shown to be a useful prognostic marker of fibrosis progression^12^. In a cohort of chronic hepatitis C patients, we found that high PRO-C3 baseline levels were associated with higher risk of fibrosis progression within 52 weeks. This further strengthens the clinical utility of PRO-C3 as a robust prognostic marker for liver fibrosis progression in both non-transplanted and transplanted patients.

It is well established that the collagen content is up to 10-fold higher in a cirrhotic than in a healthy liver^6,7^. Local tissue inflammation and damage to the epithelium result in remodeling of the underlying basement membrane^19^, monitored by type IV collagen biomarkers PRO-C4 and C4M. In the event of chronic inflammation, damage to the interstitial matrix occurs with subsequent remodeling. To replace the damaged tissue, excessive amount of interstitial types I, III and V collagens are produced by fibroblasts. The lower prognostic value of the interstitial collagen synthesis markers PRO-C3 and PRO-C5 at baseline, may reflect the fact that remodeling and excessive deposition of interstitial matrix occurs at later stages of liver fibrosis. In addition, it is notably that levels of ALT did not predict RC in this study, suggesting that the nature of RC might be repair more than inflammation. This confirms previous findings that fibrosis is not just fibrosis^19^.

The use of non-invasive diagnostic techniques, like transient elastography (Fibroscan®), has been widely accepted as alternative to biopsy to assess liver fibrosis in non-transplant patients^26–28^. TE might also be useful for monitoring post-LT patients as imaging techniques are unaffected by post-operative biochemical alterations. However, in post-LT settings TE may be affected by postoperative inflammatory reactions, rejection episodes, both of which are associated with swelling and edema that increase stiffness, hematoma, and bile duct or vascular complications^29^. Performance of TE might furthermore be comprised by the presence of ascites or obesity^30^.

It is recognized that the sample size is relatively small, which is due to the limitation of the availability of follow-up samples in this unique patient cohort. Development of advanced fibrosis and cirrhosis within 1 year is a rare event, thus limiting number of patients in the study. Nonetheless, the data provides clear results as to prediction of rapid vs intermediate vs no fibrosis progression in these patients. In this sense, this study is intended to provide the first proof-of-concept that these markers are indeed reflecting the dynamics of fibrogenesis in a unique cohort, where rapid progression to cirrhosis can occur. To our knowledge this is the first such study. It needs to be investigated whether the prognostic capacity of the markers can be repeated in larger cohorts of different etiologies of liver fibrosis given etiology, to create refined profiles of those patients likely to progress cirrhosis.

In conclusion, we have shown that remodeling of interstitial matrix is linked to clinical outcome (survival), whereas remodeling of basement membrane is rather associated with the rate of fibrosis progression. The major limitation of the study is the relatively low number of patients due to the rare event of patients that progressed to advanced fibrosis and cirrhosis within 1 year after liver transplantation. Thus, PRO-C4, C4M and PRO-C3 are proposed as clinically useful tools for prognosis, surveillance, and possibly therapy control and adjustment, in patients with progressive liver disease.

## Patients and Methods

### Patients

Caucasian patients conducted at the University of Mainz Medical Center between 1996 and 2010 were identified from a prospective transplant database.

Patients were selected based on rapidity of fibrosis progression and retrospectively divided into three groups. Those who developed histologically proven cirrhosis within 1 year (fast progressors) or within 3–5 years after LT (intermediate progressors) were compared with those who showed no or only mild fibrosis (F0/F1) in protocol biopsies taken at 5 years post-LT (non-progressors). A selection criterion was the availability of three follow up sera in the first year after LT. All patients showed no significant fibrosis of the donor organ before implantation (F0/F1). Per protocol biopsies were taken at 1- and 5-year post-LT. Hepatitis C infection (HCV) positive patients were from the era before the availability of highly effective antivirals. HCV re-infection was diagnosed by positive HCV-RNA post-LT and characteristic laboratory findings. Patients with ischemic type biliary lesions or ischemic damage of the biliary tract are summarized under “biliary” in Table 1. For execution of this study, no biological material (serum, tissue) from prisoners was used. All biological material (serum, plasma) was obtained exclusively from patients being treated and followed at Mainz University Medical Center, samples were handled in an anonymized way, and ethical approval was obtained for diagnostic and exploratory serum/plasma sampling. The study followed the ethical guidelines of the Declaration of Helsinki with signed informed consent from all patients and was approved by the local Ethics Committee of the State of Rhineland-Palatinate [no. 837.533.11(8075)].

Biochemical analysis was performed using standard routine laboratory protocols for tests including assessment of bilirubin and alanine aminotransferase (ALT). In addition, enhanced liver fibrosis (ELF) test was assessed in year 1 samples.

### Histological assessment

Liver specimens were evaluated by two experienced pathologists. The second was blinded to the results of the first pathologist. The degree of liver fibrosis was staged according to the scoring system of Desmet^31^ using a scale of 0–4 (F0: Absent; F1: mild fibrosis with periportal fibrous expansion; F2: moderate fibrosis with portal-portal septa (≥1 septum); F3: severe fibrosis with porto-central septa (≥1 septum); F4: cirrhosis).

### Immunosuppression

Patients were prescribed different immunosuppressive regiments according to individual risk factors and co-morbidities. The standard immunosuppressive regimen was the combination of a calcineurin inhibitor, either tacrolimus or cyclosporine, with mycophenolate mofetil. For tacrolimus levels were 5–7 ng/ml during the first year and 3–5 ng/ml thereafter. For cyclosporine, target trough levels were 70–90 ng/ml the first year and 50–70 ng/ml thereafter. No patients received mTOR-inhibitors, known to have potential anti-fibrotic activity^5^. All patients received steroids (methylprednisolone) until 3 months post-LT. Methylprednisolone was reduced from 1.5 mg/kg on day 1 and 2 post-LT to 1.0 mg/kg on days 3 and 4, to 0.5 mg/kg on days 5 to 14, and to 0.2 mg/kg on day 15 until 3 months post-LT. Acute rejection episodes were treated with methylprednisolone 500 mg/d i.v. for 3 days whenever a biopsy proven acute rejection occurred with a rejection activity index (RAI) ≥5.

### Collagen biomarker assessment

In this study biomarkers of interstitial type III and V collagen formation (PRO-C3^32^ and PRO-C5^16^), basement membrane type IV collagen formation (PRO-C4^8^) and MMP-2 and MMP-9 degradation (C4M^20^) were assessed by ELISAs developed by Nordic Bioscience, Herlev, Denmark. Briefly, the assays were performed as follows: 96-well pre-coated streptavidin plates (Roche Diagnostics, Mannheim, Germany) were coated with the appropriate biotinylated synthetic peptides and incubated for 30 minutes at 20 °C. Twenty µL of standard peptide or pre-diluted serum sample were added to appropriate wells, followed by peroxidase-conjugated specific monoclonal antibodies and incubated for 1 hour or overnight at 20 °C or 4 °C. Finally, tetramethylbenzidine (TMB) (cat.438OH, Kem-En-Tec Diagnostics, Taastrup, Denmark) was added, and the plates were incubated for 15 minutes at 20 °C in darkness. All the above incubation steps included shaking at 300 rpm. After each incubation step, the plate was washed five times in washing buffer (20 mM Tris, 50 mM NaCl, pH 7.2). The TMB reaction was stopped by adding 0.18 M H_2_SO_4_ as stopping solution and measured at 450 nm with 650 nm as reference. A calibration curve was plotted using a 4-parametric mathematical fit model. All samples were measured within the measurement range of each assay. Technical performance data for PRO-C3, PRO-C4, PRO-C5 and C4M can be found in Supplementary Table 1.

### Statistical analyses

Data were analyzed using MedCalc® version 14 (MedCalc Software, Ostend, Belgium). Distribution of data was found to be skewed. All correlations were calculated by Spearman rank correlation. Non-parametric continuous variables were compared between multiple groups by the Kruskal-Wallis test, p-values were adjusted for multiple comparisons. Receiver operating characteristics (ROC) curves with 95% confidence interval (CI) were calculated to determine the performance of the biomarkers. Area under the ROC (AUROC) curves were compared using the method of DeLong *et al*.^33^. Sensitivity, specificity, positive and negative predictive values and likelihood ratios were calculated at thresholds derived from ROC curves. Serum levels of the biomarkers were divided into tertiles, to calculate the odds ratios (OR) for progression towards recurrent cirrhosis (RC). Tertiles were chosen due to the small number of patients. The lowest tertile was used as reference. Univariate analyses on patient’s characteristics as predictors of mortality were calculated by Logistic regression. Patient survival was calculated by Kaplan-Meier analysis, and survival curves were compared using Log-rank test.

Graphs were designed using GraphPad Prism® version 7 (GraphPad Software, Inc., La Jolla, CA, USA). Data are shown as Tukey box plots. Dotted lines represent reference level from normal population. P-values less than 0.05 were considered statistically significant, given as *p < 0.05; **p < 0.01 and ***p < 0.001.

## Supplementary information

.Supplementary information
