# Supplementary material for: Serum markers of type III and IV procollagen processing predict recurrence of fibrosis in liver transplanted patients
Source: Sci Rep. 2019 Oct 16;9:14857. doi: 10.1038/s41598-019-51394-4 (PMC6796007; doi:10.1038/s41598-019-51394-4)

**Supplementary material**

**Serum markers of type III and IV procollagen processing predict recurrence of fibrosis in liver transplanted patients.**

**Short Title:** Fibrosis markers of liver transplant patients

^1^Mette Juul Nielsen^¤,^**^*^**, ^1^Ida Falk Villesen**^¤^**, ^1^Natasja Stæhr Gudmann, ^1^Diana Julie Leeming, ^3^Aleksander Krag, ^1^Morten Asser Karsdal, ^2^Tim Zimmermann**^¤^** and ^4,5^Detlef Schuppan^¤^

^1^Nordic Bioscience, Fibrosis Biology and Biomarkers, Herlev, Denmark

^2^Department of Medicine I, Transplant Hepatology, University Medical Center, Mainz, Germany

^3^Department of Gastroenterology and Hepatology, Odense University Hospital, University of Southern Denmark, Odense, Denmark

^4^Institute of Translational Immunology, University Medical Center, Mainz, Germany

^5^Division of Gastroenterology, Beth Israel Deaconess Medical Center, Harvard Medical School, Boston, MA

**¤** Authors that contributed equally

**Supplementary table 1:** Technical performances of PRO-C3, PRO-C4, PRO-C5, C4M. Other technical performances including specificity and stability are described in references 8, 16, 24 and 25.

| Technical parameter | PRO-C3 | PRO-C4 | PRO-C5 | C4M |
| --- | --- | --- | --- | --- |
| IC50, range ng/mL | 5.5 – 8.5 | 37.4 – 67.3 | 80.0 – 112.0 | 5.0 – 7.0 |
| LLOD, ng/mL | 1.1 | 0.5 | 4.5 | 0.7 |
| Measurement range (LLOQ-ULOQ), ng/mL | 1.3 – 58.0 | 6.0 – 348.5 | 32.5 – 1189.0 | 2.2 – 72.0 |
| Intra-assay variation, range | 1.5 – 8.3% | 2.8 – 5.8% | 2.0 – 9.0% | 4.0 – 10.0 |
| Inter-assay variation, range | 3.6 – 13.3% | 5.2 – 17.6% | 5.0 – 15.0% | 5.0 – 15.0 |
| Linearity, %RE | -8.1 – 17.3% | -8.2 – 12.0% | -1.6% – 7.4% | 8.0 – 18.0 |
| Reference range*, mean ng/mL (95% CI) | 12.1  (6.7-22.2) | 153.6  (74.9-281.2) | 394.5  (207.4-611.7) | 21.4  (19.4-23.3) |

*reference ranges are corrected for predilution of samples assessed in serum from a healthy population. LLOD: Lower limit of detection; LLOQ: Lower limit of quantification; ULOQ: Upper limit of quantification.

**Supplementary Table 2**. Spearman rank correlation coefficient for the ELF test and its components at 1 year after liver transplantation; ELF, HA, TIMP, PIIINP and serum markers; C4M, PRO-C3, PRO-C4 and PRO-C5 one year after liver transplant. Significance >0.05 is indicated in bold.

|  | | C4M | PRO-C3 | PRO-C5 | PRO-C4 |
| --- | --- | --- | --- | --- | --- |
| ELF | Correlation coefficient | 0.056 | 0.793 | 0.19 | 0.098 |
|  | Significance Level P | 0.7205 | **<0.0001** | 0.222 | 0.5302 |
|  | n | 43 | 43 | 43 | 43 |
| HA | Correlation coefficient | -0.037 | 0.529 | 0.159 | 0 |
|  | Significance Level P | 0.8115 | **0.0003** | 0.3094 | 0.9992 |
|  | n | 43 | 43 | 43 | 43 |
| TIMP-1 | Correlation coefficient | 0.331 | 0.808 | 0.406 | 0.372 |
|  | Significance Level P | **0.0284** | **<0.0001** | **0.0063** | **0.013** |
|  | n | 44 | 44 | 44 | 44 |
| PIIINP | Correlation coefficient | 0.178 | 0.905 | 0.198 | 0.254 |
|  | Significance Level P | 0.2488 | **<0.0001** | 0.1969 | 0.0968 |
|  | n | 44 | 44 | 44 | 44 |

**Supplementary Table 3**. Spearman rank correlation coefficients for the clinical parameters at 3 months, 6 months, 1 year, 2 years and 3 years after liver transplantation; Albumin, ALT, AP, AST, Bilirubin, gGT, INR, Quick%, and platelets versus serum markers; C4M, PRO-C4, PRO-C3 and PRO-C5. Significance >0.05 is indicated in bold. ALT: Alanine aminotransferase, AP: Alkaline phosphatase; gGT: gamma-glutamyltransferase; INR: international normalized ratio. N, 3 months=19-29; N, 6 months=15-30; N 1 year=31-44, N, 2 years=27-33; N, 3 years=30-36.

|  | | | Albumin | | ALT | | AP | AST | | Bilirubin | | | gGT | | INR | Quick | | Platelets | |
| --- | --- | --- | --- | --- | --- | --- | --- | --- | --- | --- | --- | --- | --- | --- | --- | --- | --- | --- | --- |
| 3 months after liver transplantation | | |  | |  | |  |  | |  | | |  | |  |  | |  | |
| Mean (95% CI) | | | 40.2 (38.1-42.4) | | 107 (51-163.2) | | 354.7 (220.3-489) | 76 (30.9-121.2) | | 1.5 (1-2) | | | 295.8 (154-437.6) | | 1.1 (1-1.1) | 92.1 (88.1-96.1) | | 162.4 (139.7-185.1) | |
| C4M | Correlation coefficient P-value | | -0.512 **0.0075** | | 0.124 0.5285 | | 0.174 0.3772 | 0.316 0.1014 | | 0.164 0.4046 | | | 0.298 0.1234 | | 0.115 0.6283 | -0.158 0.4322 | | 0.206 0.3031 | |
| PRO-C4 | Correlation coefficient P-value | | -0.548 **0.0038** | | 0.168 0.3848 | | 0.103 0.5951 | **0.412 0.0293** | | 0.143 0.4607 | | | 0.217 0.2581 | | 0.302 0.1957 | -0.248 0.2126 | | 0.056 0.7756 | |
| PRO-C3 | Correlation coefficient P-value | | -0.483 **0.0144** | | -0.032 0.8750 | | -0.042 0.8371 | 0.154 0.4528 | | -0.008 0.9674 | | | 0.202 0.3120 | | 0.031 0.8999 | -0.019 0.9273 | | -0.037 0.8578 | |
| PRO-C5 | Correlation coefficient P-value | | -0.434 **0.0267** | | 0.074 0.7030 | | 0.144 0.4551 | 0.273 0.1598 | | 0.208 0.2797 | | | 0.115 0.5532 | | **0.525 0.0174** | -0.327 0.0960 | | -0.119 0.5480 | |
| 6 months after liver transplantation | | |  | |  | |  |  | |  | | |  | |  |  | |  | |
| Mean (95% CI) | | | 40.3 (38.5-42) | | 62.8 (46.2-79.4) | | 324.9 (225.5-424.4) | 48.9 (37.4-60.4) | | 1.3 (0.9-1.6) | | | 408.5 (257.2-559.7) | | 1 (1-1.1) | 98 (90.6-105.2) | | 162.3 (137.6-187.1) | |
| C4M | Correlation coefficient P-value | | -0.668 **0.0003** | | 0.183 0.3809 | | -0.089 0.6714 | 0.370 0.0630 | | 0.226 0.2765 | | | 0.214 0.2930 | | 0.039 0.8822 | -0.046 0.8263 | | -0.055 0.7939 | |
| PRO-C4 | Correlation coefficient P-value | | -0.570 **0.0024** | | -0.069 0.7386 | | -0.145 0.4715 | 0.115 0.5601 | | 0.106 0.6049 | | | 0.003 0.9868 | | 0.057 0.8228 | -0.059 0.7763 | | 0.004 0.9854 | |
| PRO-C3 | Correlation coefficient P-value | | -0.191 0.3819 | | -0.144 0.5126 | | -0.212 0.3304 | 0.105 0.6259 | | 0.246 0.2581 | | | -0.305 0.1470 | | -0.163 0.5607 | -0.040 0.8577 | | -0.052 0.8124 | |
| PRO-C5 | Correlation coefficient P-value | | -0.528 **0.0055** | | -0.128 0.5335 | | -0.208 0.2974 | 0.083 0.6737 | | 0.093 0.6499 | | | -0.085 0.6657 | | 0.005 0.9835 | -0.015 0.9418 | | -0.044 0.8293 | |
| 1 year after liver transplantation | |  | |  | |  | | |  | |  |  | |  | | |  | |  |
| Mean (95% CI) | | 40 (38-41.8) | | 75.3 (48-102.7) | | 498 (109.6-886.3) | | | 65.4 (44.7-86.2) | | 2.6 (0.2-5) | 375 (149.2-600) | | 1.1 (0.9-1.4) | | | 96.6 (91.3-102) | | 163 (136.6-189.4) |
| C4M | Correlation coefficient P-value | -0.330 0.0526 | | -0.017 0.9158 | | 0.362 **0.0237** | | | 0.108 0.5082 | | 0.192 0.2552 | 0.175 0.2805 | | 0.187 0.3321 | | | -0.257 0.1303 | | 0.135 0.4192 |
| PRO-C4 | Correlation coefficient P-value | -0.235 0.1740 | | 0.038 0.8177 | | 0.326 **0.0431** | | | 0.238 0.1389 | | 0.083 0.6237 | 0.091 0.5746 | | 0.249 0.1932 | | | -0.291 0.0854 | | 0.020 0.9035 |
| PRO-C3 | Correlation coefficient P-value | -0.510 **0.0017** | | 0.262 0.1026 | | 0.376 **0.0184** | | | 0.510 **0.0008** | | 0.412 **0.0112** | 0.506 **0.0009** | | 0.434 **0.0185** | | | -0.386 **0.0201** | | -0.173 0.2980 |
| PRO-C5 | Correlation coefficient P-value | -0.237 0.1698 | | -0.095 0.5596 | | 0.249 0.1257 | | | 0.129 0.4278 | | 0.178 0.2921 | 0.086 0.5964 | | 0.160 0.4065 | | | -0.371 **0.0261** | | 0.091 0.5849 |
| 2 years after liver transplantation | |  | |  | |  | | |  | |  |  | |  | | |  | |  |
| Mean (95% CI) | | 40.1 (38.1-42.1) | | 55.9 (38.2-73.6) | | 286.3 (173.6-399) | | | 55.6 (38.4-72.8) | | 1.2 (0.9-1.5) | 260.3 (155.6-365) | | 7.3 (-1.5-16.1) | | | 101 (95.2-106.7) | | 158.5 (133.8-183.3) |
| C4M | Correlation coefficient P-value | -0.483 **0.0092** | | 0.064 0.7326 | | 0.314 0.0850 | | | 0.399 **0.0291** | | 0.304 0.1095 | 0.408 **0.0204** | | -0.237 0.2346 | | | -0.020 0.9145 | | 0.247 0.1879 |
| PRO-C4 | Correlation coefficient P-value | -0.417 **0.0271** | | 0.076 0.6851 | | 0.328 0.0712 | | | 0.424 **0.0195** | | 0.204 0.2873 | 0.367 **0.0388** | | -0.185 0.3547 | | | -0.076 0.6885 | | 0.342 0.0647 |
| PRO-C3 | Correlation coefficient P-value | -0.612 **0.0005** | | 0.396 **0.0274** | | 0.566 **0.0009** | | | 0.637 **0.0002** | | 0.591 **0.0007** | 0.622 **0.0001** | | -0.008 0.9672 | | | -0.151 0.4247 | | -0.415 **0.0226** |
| PRO-C5 | Correlation coefficient P-value | -0.447 **0.0171** | | 0.102 0.5834 | | 0.161 0.3871 | | | 0.425 **0.0192** | | 0.192 0.3186 | 0.332 0.0637 | | -0.277 0.1615 | | | 0.026 0.8923 | | 0.229 0.2241 |
| 3 years after liver transplantation | |  | |  | |  | | |  | |  |  | |  | | |  | |  |
| Mean (95% CI) | | 41.1 (38.5-43.6) | | 47.9 (38-57.9) | | 254.8 (132.5-377.1) | | | 54.1 (38.6-69.7) | | 1.4 (0.8-2) | 220.6 (131.4-309.8) | | 1.2 (0.8-1.5) | | | 97.1 (89.9-104.3) | | 172.6 (148.9-196.2) |
| C4M | Correlation coefficient P-value | -0.387 **0.0345** | | 0.035 0.8477 | | 0.549 **0.0014** | | | 0.155 0.3886 | | -0.135 0.4522 | 0.429 **0.0160** | | 0.210 0.2661 | | | -0.241 0.1999 | | 0.138 0.4679 |
| PRO-C4 | Correlation coefficient P-value | -0.250 0.1820 | | -0.003 0.9853 | | 0.439 **0.0134** | | | 0.117 0.5166 | | -0.240 0.1785 | 0.348 0.0553 | | 0.100 0.5985 | | | -0.194 0.3041 | | 0.186 0.3238 |
| PRO-C3 | Correlation coefficient P-value | -0.591 **0.0006** | | 0.242 0.1747 | | 0.674 **<0.0001** | | | 0.502 **0.0029** | | 0.411 **0.0176** | 0.497 **0.0045** | | 0.421 **0.0206** | | | -0.343 0.0632 | | -0.310 0.0950 |
| PRO-C5 | Correlation coefficient P-value | -0.330 0.0748 | | 0.084 0.6411 | | 0.453 **0.0104** | | | 0.224 0.2108 | | -0.082 0.6489 | 0.392 **0.0293** | | 0.195 0.3028 | | | -0.241 0.2003 | | 0.099 0.6026 |

**Supplementary table 4:** Univariate and multivariate cox regression analyses for prediction of recurrent cirrhosis 1 year after liver transplantation using blood samples taken 3 months after transplantation.

|  | P value | Hazard ratio | 95% CI |
| --- | --- | --- | --- |
| *Univariate* |  |  |  |
| Albumin | ***0.0139*** | 0.8701 | 0.7788 to 0.9721 |
| ALT | 0.9575 | 0.9999 | 0.9956 to 1.0041 |
| AP | 0.3205 | 0.9986 | 0.9957 to 1.0014 |
| AST | 0.9274 | 0.9997 | 0.9943 to 1.0052 |
| bilirubin | 0.1817 | 1.2926 | 0.8869 to 1.8840 |
| ggt | 0.6091 | 1.0004 | 0.9990 to 1.0017 |
| inr | 0.4202 | 57.8129 | 0.0030 to 1.11x10^6^ |
| quick | 0.1812 | 0.9569 | 0.8972 to 1.0207 |
| platelets | 0.1023 | 1.0098 | 0.9981 to 1.0217 |
| PRO-C3 | ***0.0011*** | 1.0274 | 1.0108 to 1.0442 |
| PRO-C4 | ***0.0004*** | 1.0087 | 1.0038 to 1.0135 |
| PRO-C5 | 0.0598 | 1.0029 | 0.9999 to 1.0060 |
| C4M | ***0.0008*** | 1.1488 | 1.0590 to 1.2461 |
| *Multivariate* |  |  |  |
| PRO-c3 | ***0.0317*** | 1.0194 | 1.0017 to 1.0374 |
| PRO-C4 | ***0.0473*** | 1.0051 | 1.0001 to 1.0103 |

**Supplementary table 5:** Univariate and multivariate cox regression analyses for prediction of recurrent cirrhosis 1 year after liver transplantation using blood samples taken 6 months after transplantation.

|  | P value | Hazard ratio | 95% CI |
| --- | --- | --- | --- |
| *Univariate* |  |  |  |
| Albumin | ***0.0037*** | 0.8030 | 0.6923 to 0.9312 |
| ALT | 0.8069 | 1.0023 | 0.9838 to 1.0213 |
| AP | 0.1621 | 0.9973 | 0.9936 to 1.0011 |
| AST | ***0.0464*** | 1.0226 | 1.0004 to 1.0453 |
| bilirubin | ***0.0138*** | 2.3053 | 1.1861 to 4.4806 |
| ggt | 0.5039 | 1.0005 | 0.9989 to 1.0021 |
| inr | 0.0601 | 552.0882 | 0.7642 to 3.99x10^5^ |
| quick | 0.0176 | 0.9623 | 0.9322 to 0.9933 |
| platelets | 0.9016 | 1.0008 | 0.9886 to 1.0131 |
| PRO-C3 | 0.0821 | 1.0110 | 0.9986 to 1.0236 |
| PRO-C4 | ***0.0002*** | 1.0058 | 1.0027 to 1.0089 |
| PRO-C5 | ***0.0012*** | 1.0028 | 1.0011 to 1.0044 |
| C4M | ***0.0002*** | 1.1020 | 1.0479 to 1.1588 |
| *Multivariate* |  |  |  |
| PRO-c5 | ***0.0140*** | 0.9897 | 0.9815 to 0.9979 |
| C4M | ***0.0003*** | 1.3937 | 1.1632 to 1.6699 |

**Supplementary table 6:** Univariate and multivariate cox regression analyses for prediction of recurrent cirrhosis 3-5 years after liver transplantation using blood samples taken 1 year after transplantation.

|  | P value | Hazard ratio | 95% CI |
| --- | --- | --- | --- |
| *Univariate* |  |  |  |
| Albumin | ***0.0168*** | 0.9197 | 0.8587 to 0.9850 |
| ALT | 0.1217 | 1.0036 | 0.9991 to 1.0081 |
| AP | 0.2751 | 1.0002 | 0.9999 to 1.0004 |
| AST | ***0.0266*** | 1.0058 | 1.0007 to 1.0110 |
| bilirubin | ***0.0257*** | 1.0471 | 1.0056 to 1.0903 |
| ggt | ***0.0189*** | 1.0005 | 1.0001 to 1.0009 |
| inr | 0.4402 | 0.5425 | 0.1148 to 2.5643 |
| quick | 0.1638 | 0.9810 | 0.9550 to 1.0078 |
| platelets | 0.4796 | 0.9978 | 0.9915 to 1.0040 |
| PRO-C3 | ***0.0002*** | 1.0229 | 1.0108 to 1.0350 |
| PRO-C4 | 0.5925 | 1.0004 | 0.9990 to 1.0018 |
| PRO-C5 | 0.1101 | 1.0012 | 0.9997 to 1.0026 |
| C4M | 0.3554 | 1.0136 | 0.9850 to 1.0429 |
| *Multivariate* |  |  |  |
| PRO-c3 | ***0.0079*** | 1.0178 | 1.0046 to 1.0311 |

**Supplementary table 7:** Univariate and multivariate cox regression analyses for prediction of recurrent cirrhosis 3-5 years after liver transplantation using blood samples taken 2 year after transplantation.

|  | P value | Hazard ratio | 95% CI |
| --- | --- | --- | --- |
| *Univariate* |  |  |  |
| Albumin | ***0.0136*** | 0.8959 | 0.8210 to 0.9777 |
| ALT | 0.0935 | 1.0074 | 0.9988 to 1.0162 |
| AP | ***0.0130*** | 1.0017 | 1.0004 to 1.0030 |
| AST | 0.0505 | 1.0071 | 1.0000 to 1.0142 |
| bilirubin | ***0.0007*** | 2.3371 | 1.4289 to 3.8225 |
| ggt | ***0.0280*** | 1.0012 | 1.0001 to 1.0023 |
| inr | 0.9970 | 1.0000 | 0.9778 to 1.0226 |
| quick | 0.2038 | 0.9820 | 0.9548 to 1.0099 |
| platelets | 0.7414 | 0.9985 | 0.9898 to 1.0073 |
| PRO-C3 | ***0.0002*** | 1.0337 | 1.0160 to 1.0518 |
| PRO-C4 | 0.3310 | 1.0007 | 0.9993 to 1.0021 |
| PRO-C5 | ***0.0248*** | 1.0021 | 1.0003 to 1.0040 |
| C4M | ***0.0144*** | 1.0494 | 1.0097 to 1.0907 |
| *Multivariate* |  |  |  |
| PRO-c3 | **0.0006** | 1.0357 | 1.0151 to 1.0567 |
| PRO-C5 | **0.0070** | 1.0034 | 1.0009 to 1.0058 |

**Supplementary table 8:** Univariate and multivariate cox regression analyses for prediction of recurrent cirrhosis 3-5 years after liver transplantation using blood samples taken 3 year after transplantation.

|  | P value | Hazard ratio | 95% CI |
| --- | --- | --- | --- |
| *Univariate* |  |  |  |
| Albumin | ***<0.0001*** | 0.8620 | 0.8028 to 0.9255 |
| ALT | 0.2998 | 1.0071 | 0.9937 to 1.0208 |
| AP | ***0.0010*** | 1.0020 | 1.0008 to 1.0032 |
| AST | ***0.0001*** | 1.0199 | 1.0097 to 1.0302 |
| bilirubin | ***0.0053*** | 1.2836 | 1.0771 to 1.5297 |
| ggt | ***0.0030*** | 1.0022 | 1.0007 to 1.0037 |
| inr | 0.5825 | 0.7752 | 0.3126 to 1.9220 |
| quick | 0.5735 | 0.9944 | 0.9750 to 1.0141 |
| platelets | 0.1799 | 0.9935 | 0.9841 to 1.0030 |
| PRO-C3 | ***0.0007*** | 1.0152 | 1.0064 to 1.0241 |
| PRO-C4 | 0.3057 | 1.0008 | 0.9993 to 1.0022 |
| PRO-C5 | 0.1307 | 1.0009 | 0.9997 to 1.0021 |
| C4M | 0.0656 | 1.0256 | 0.9984 to 1.0535 |
| *Multivariate* |  |  |  |
| PRO-c3 | ***0.0125*** | 1.0134 | 1.0029 to 1.0241 |
| Albumin | ***0.0003*** | 0.8710 | 0.8077 to 0.9391 |

**Supplementary table 9:** Univariate cox regression analyses for prediction of recurrent cirrhosis in both fast and intermediate progressors after liver transplantation using biomarker ∆ levels from baseline to 6 months 1, 2 or 3 years after transplantation.

|  | P value | Hazard ratio | 95% CI |
| --- | --- | --- | --- |
| *Univariate ∆6mon* |  |  |  |
| PRO-C3 | 0.0596 | 0.9808 | 0.9612 to 1.0008 |
| PRO-C4 | ***0.0078*** | 1.0074 | 1.0019 to 1.0129 |
| C4M | 0.2278 | 1.0315 | 0.9808 to 1.0848 |
| PRO-C5 | 0.0763 | 1.0024 | 0.9997 to 1.0050 |
| *Univariate ∆1y* |  |  |  |
| PRO-C3 | 0.7739 | 1.0023 | 0.9866 to 1.0182 |
| PRO-C4 | 0.6446 | 0.9995 | 0.9972 to 1.0017 |
| C4M | 0.0715 | 0.9497 | 0.8978 to 1.0045 |
| PRO-C5 | 0.9640 | 0.999 | 0.9973 to 1.0026 |
| *Univariate ∆2y* |  |  |  |
| PRO-C3 | 0.0930 | 1.0197 | 0.9967 to 1.0432 |
| PRO-C4 | 0.4829 | 1.0008 | 0.9986 to 1.0029 |
| C4M | 0.1741 | 1.0629 | 0.9734 to 1.1607 |
| PRO-C5 | 0.0855 | 1.0043 | 0.9994 to 1.0091 |
| *Univariate ∆3y* |  |  |  |
| PRO-C3 | 0.1842 | 1.0155 | 0.9927 to 1.0389 |
| PRO-C4 | 0.9531 | 1.0001 | 0.9975 to 1.0027 |
| C4M | 0.8387 | 1.0059 | 0.9507 to 1.0642 |
| PRO-C5 | 0.8305 | 1.0003 | 0.9975 to 1.0031 |

**Supplementary figure 1:** Tukey Boxplots of biomarker levels at 1 year after liver transplantation for PRO-C3, PRO-C4, C4M and PRO-C5. Patients were stratified according to their progression rate towards cirrhosis after LT.


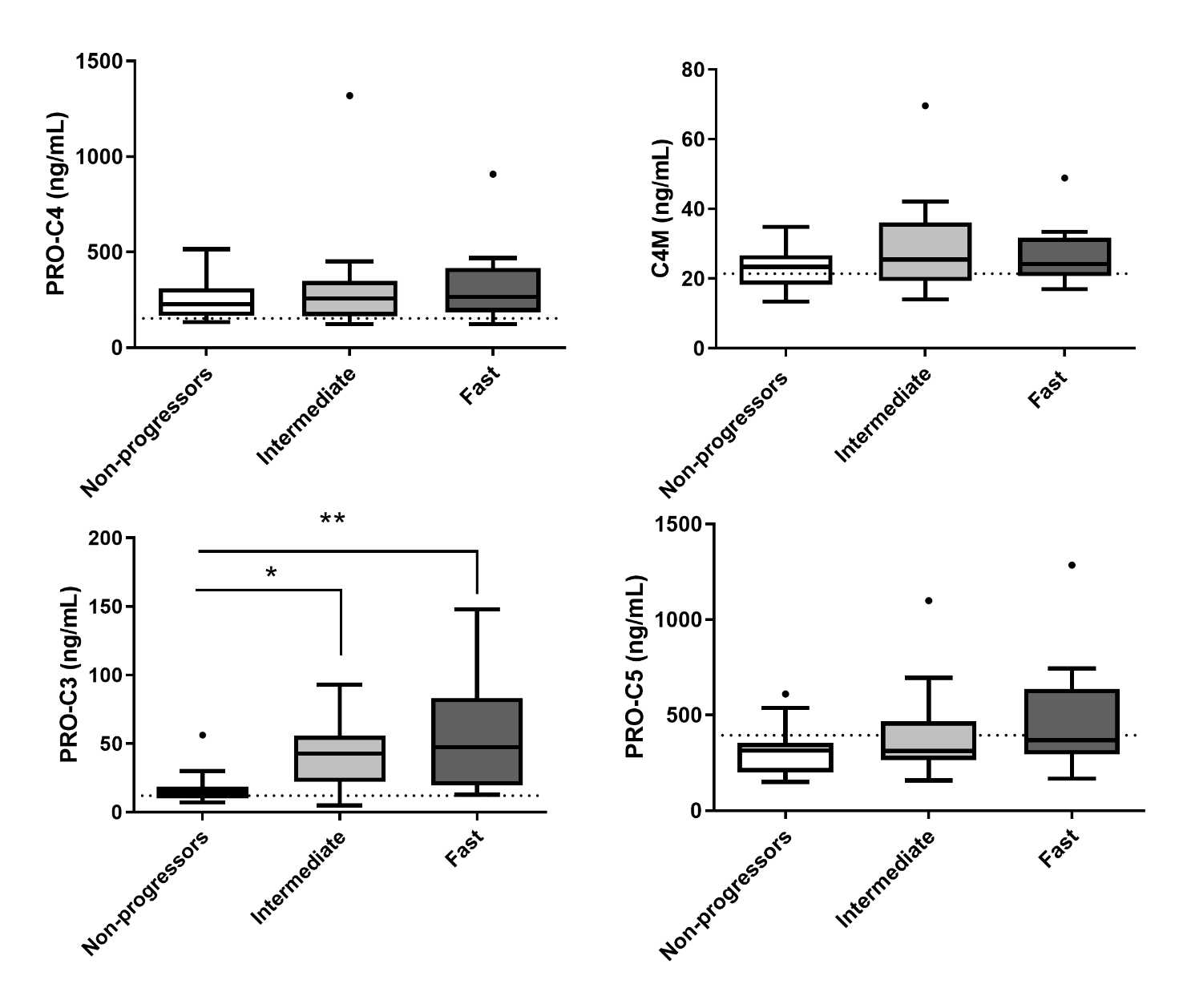


**Supplementary figure 2:** Tukey Boxplots of biomarker levels at 2 years after liver transplantation for PRO-C3, PRO-C4, C4M and PRO-C5. Patients were stratified according to their progression rate towards cirrhosis after LT.


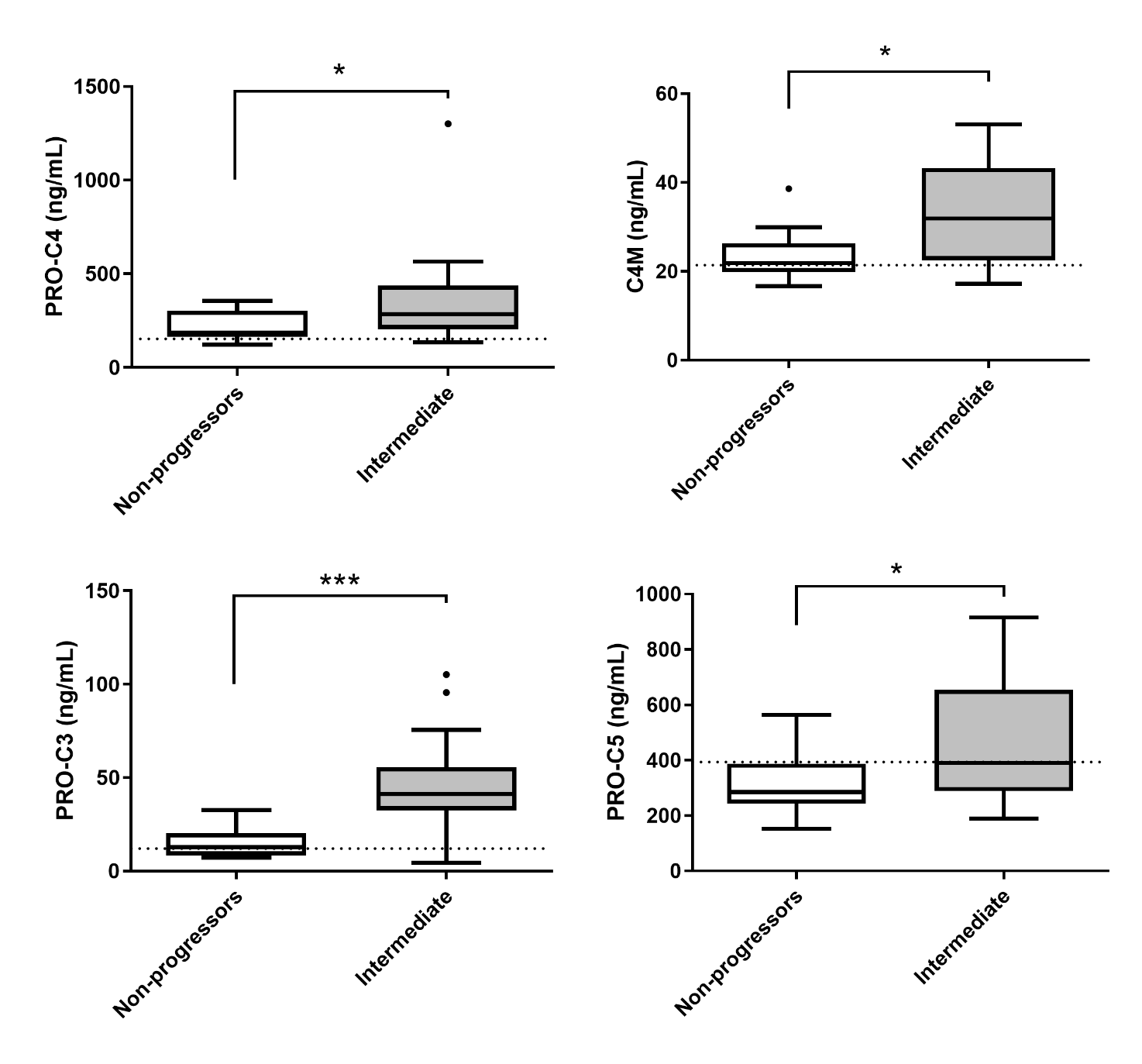


**Supplementary figure 3:** Tukey Boxplots of biomarker levels at 3 years after liver transplantation for PRO-C3, PRO-C4, C4M and PRO-C5. Patients were stratified according to their progression rate towards cirrhosis after LT.


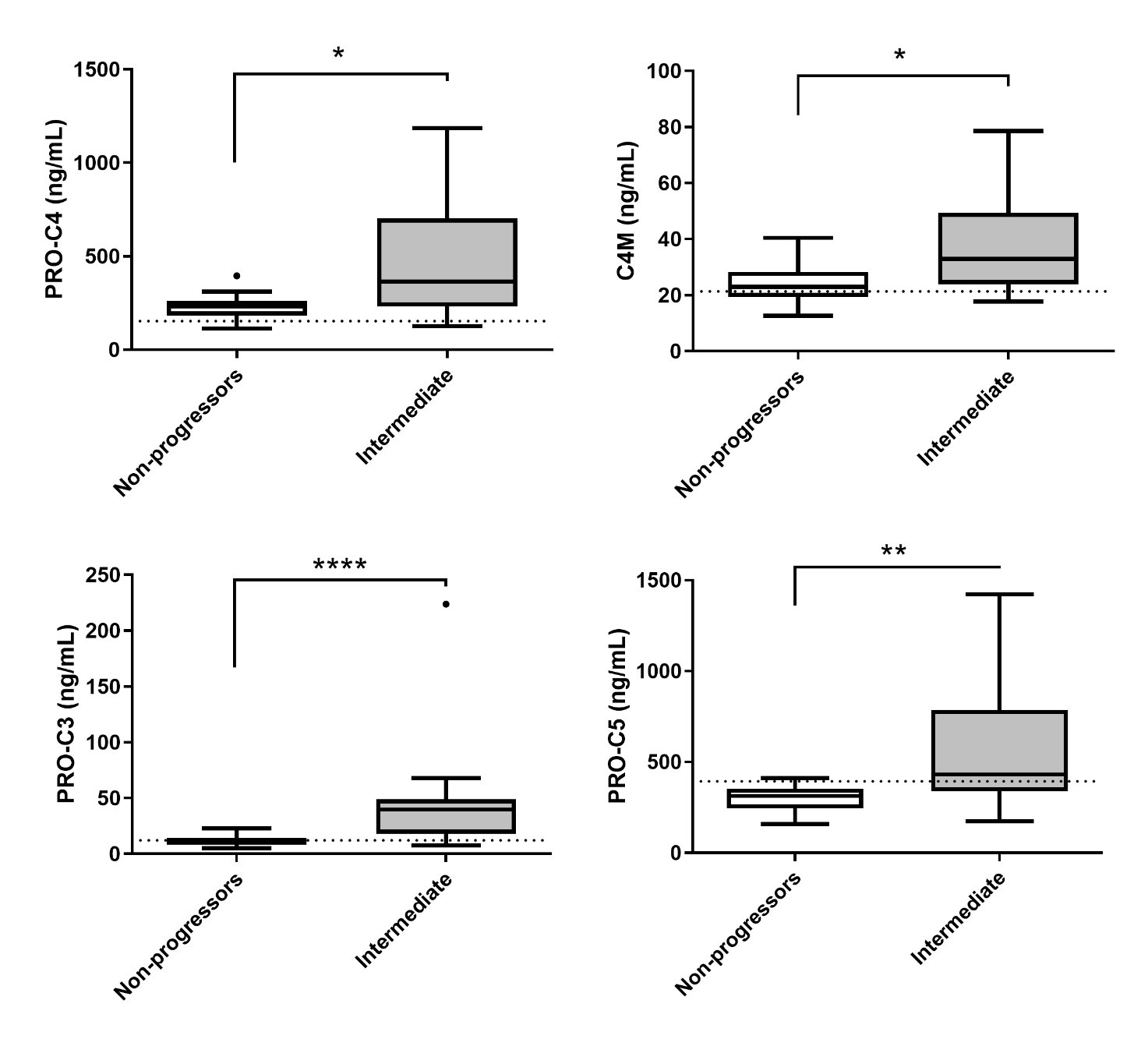


**Supplementary figure 4:** Kinetics of biomarker levels over time after liver transplantation for PRO-C3, PRO-C4, C4M and PRO-C5. Patients were stratified according to their progression rate towards cirrhosis after LT.


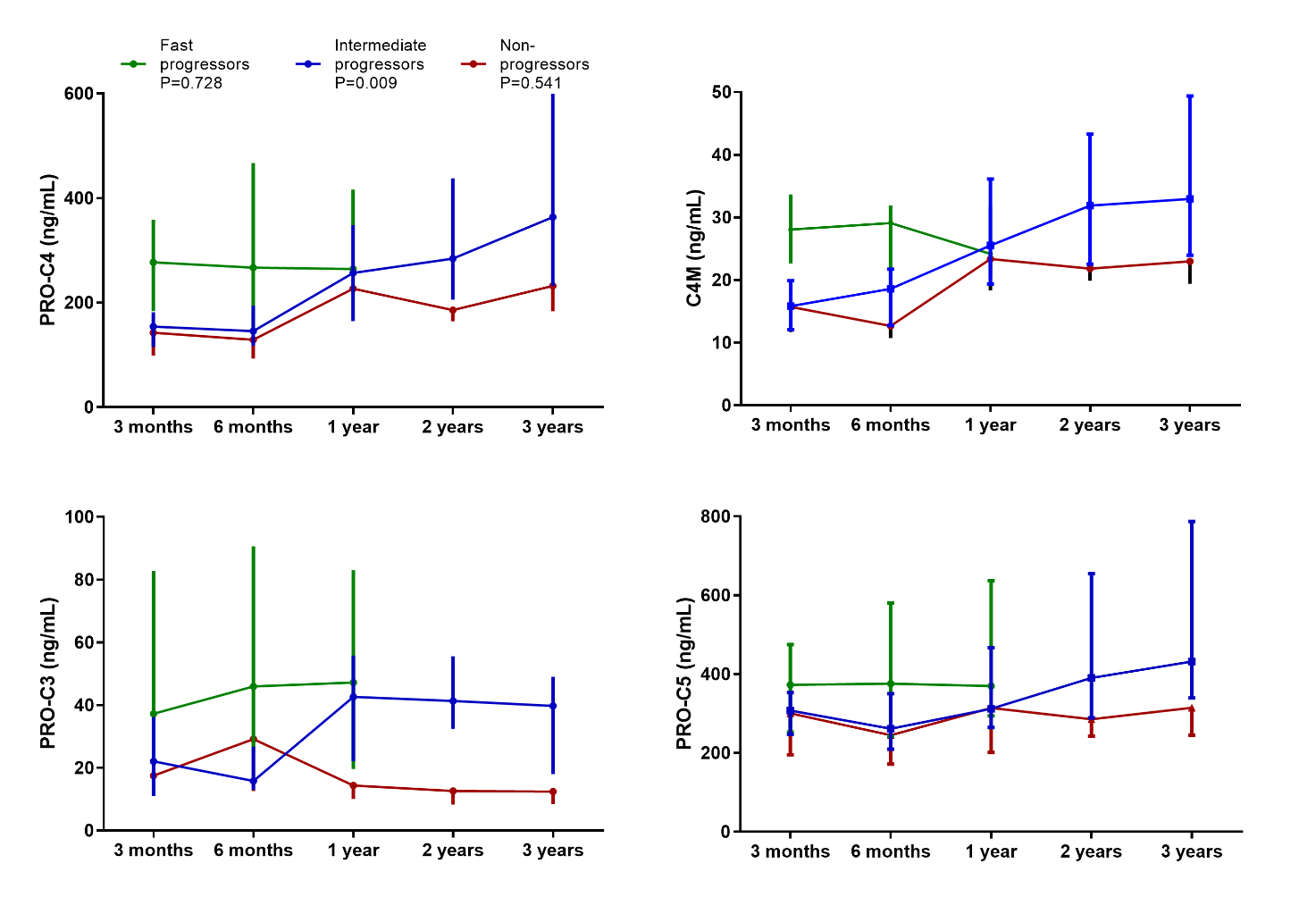


**Supplementary figure 5:** Biomarker levels over time for every patient after liver transplantation for PRO-C3, PRO-C4, C4M and PRO-C5. Patients were stratified according to their progression rate towards cirrhosis after LT.


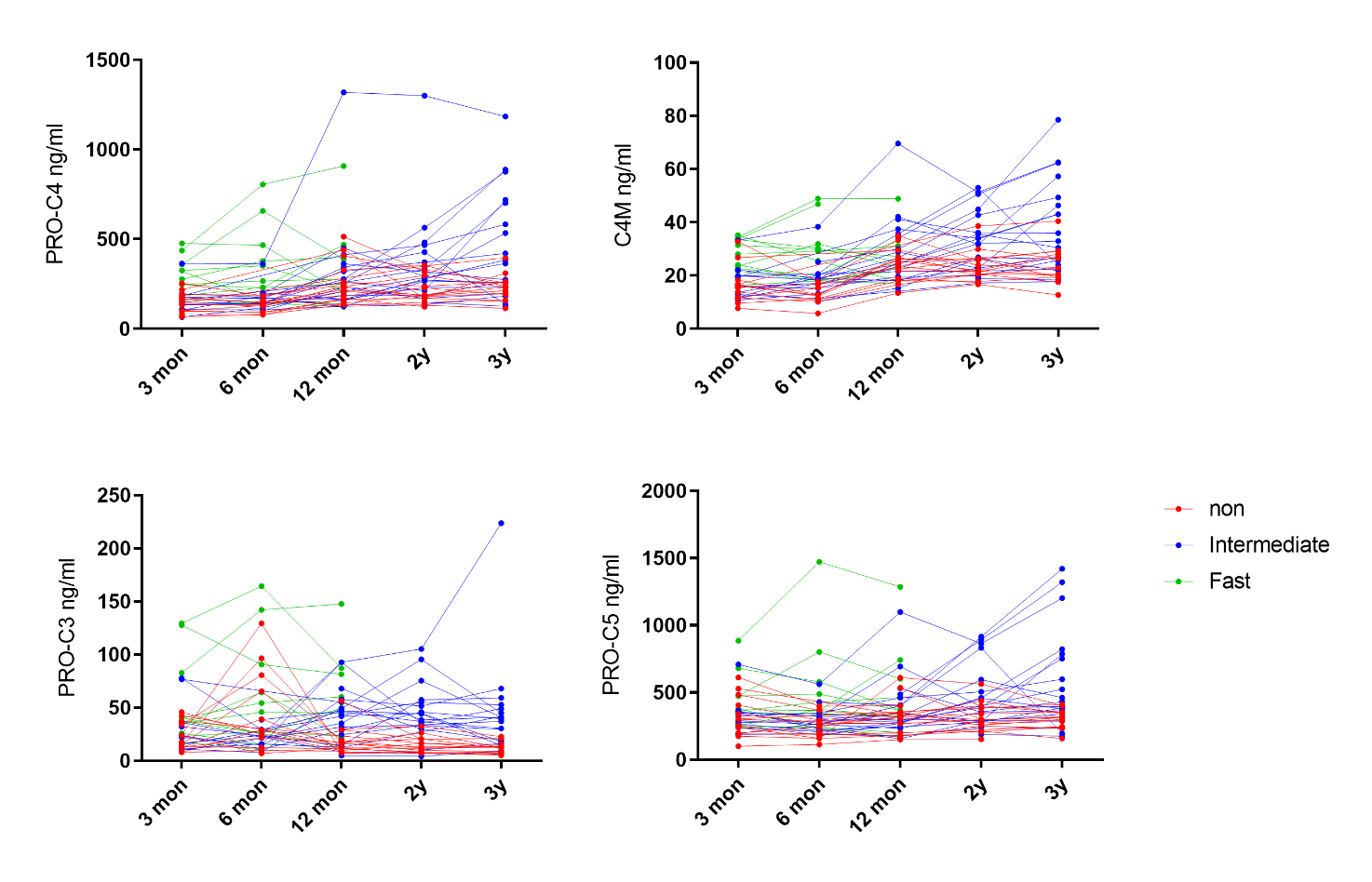

Supplement: Supplementary file 1 — .Supplementary information [file 41598_2019_51394_MOESM1_ESM.docx]
